# Environmental *Burkholderia cepacia* Complex Isolates from Human Infections

**DOI:** 10.3201/eid1303.060403

**Published:** 2007-03

**Authors:** Adam Baldwin, Eshwar Mahenthiralingam, Pavel Drevinek, Peter Vandamme, John R. Govan, David J. Waine, John J. LiPuma, Luigi Chiarini, Claudia Dalmastri, Deborah A. Henry, David P. Speert, David Honeybourne, Martin C. J. Maiden, Chris G. Dowson

**Affiliations:** * Warwick University, Coventry, Wales, United Kingdom; † Cardiff University, Cardiff, England, United Kingdom; ‡ Universiteit Gent, Ghent, Belgium; § University of Edinburgh Medical School, Edinburgh, Scotland, United Kingdom; ¶ University of Michigan Medical School, Ann Arbor, Michigan, USA; # Ente per le Nuove Tecnologie l’Energia e l’Ambiente Casaccia, Rome, Italy; ** University of British Columbia, Vancouver, British Columbia, Canada; †† Birmingham Heartlands Hospital, Birmingham, England, United Kingdom; ‡‡ University of Oxford, Oxford, England, United Kingdom

**Keywords:** MLST, *Burkholderia cepacia* complex, epidemiology, identical, genotype, environmental, clinical, global, widespread, dispatch

## Abstract

Members of the *Burkholderia cepacia* complex (Bcc), found in many environments, are associated with clinical infections. Examining diverse species and strains from different environments with multilocus sequence typing, we identified >20% of 381 clinical isolates as indistinguishable from those in the environment. This finding links the natural environment with the emergence of many Bcc infections.

The *Burkholderia cepacia* complex (Bcc) is a group of closely related gram-negative bacteria comprising at least 9 species (*1*). They are routinely isolated from the natural environment, where they can have a range of beneficial properties (*2*). However, these bacteria can also frequently cause fatal infections in vulnerable humans, such as those who have cystic fibrosis (CF). Because Bcc bacteria are not normally carried as commensal organisms, the main sources of infection are considered to be patient-to-patient spread (*3*,*4*); hospital settings, including medical devices and contaminated disinfectants; and the environment (*5*,*6*). Therefore, although Bcc species may have an important environmental role in agriculture and biotechnology industries, their use also represents a potential clinical risk to susceptible members of the community (*7**,**8*). All species of Bcc can be isolated from the environment in differing degrees (*2*). Similarly, all current Bcc species have been cultured from CF patient sputum (*2*). Infection control measures have been implemented to reduce patient-to-patient transmission; although effective, these measures have not prevented the emergence of new infection. Thus, the environment could be acting as a constant nonpatient reservoir for infectious Bcc pathogens.

Previous studies have reported the possibility of humans acquiring Bcc directly from natural environments (*9*,*10*). The most recent of these studies reported evidence that a *B. cenocepacia* strain, isolated from soil, was indistinguishable by several typing methods (pulsed-field gel electrophoresis [PFGE] genomic fingerprinting and repetitive extragenic palindromic [rep]–PCR) from isolates of the problematic CF lineage PHDC (*10*).

## The Study

To evaluate how widespread the emergence of environmental isolates as causes of clinical infections may be, we used a highly discriminatory and transportable typing method to study isolates from several large bacterial culture collections. Multilocus sequence typing (MLST) is a relatively new technique based upon unambiguous sequence analysis of several housekeeping genes. Unlike previous methods for Bcc strain typing (*10*), MLST is not based on banding patterns but instead relies on the robust comparison of DNA sequence information. This process facilitates both the identification and matching of identical clones as well as their evolutionary comparison to closely related strains.

Using a recently validated MLST scheme (*11*), we analyzed a collection of 381 clinical isolates of all 9 currently reported Bcc species, from 28 countries. A total of 187 distinct sequence types (STs) were identified from clinical isolates within this collection and compared with 233 environmental Bcc isolates (113 STs). We found that 81 clinical isolates (encompassing15 STs) were identical by MLST to a wide range of environmental isolates. This figure represents 21.5% of the clinical isolates examined (for clarity, a subset are shown in the Table; [*12*]).

**Table:** MLST analysis of the *Burkholderia cepacia* strains showing their species, source, and geographic location*

Bcc species	ST	Isolate name	Source	Country	Source of isolate:	Year of isolation
*B. cepacia*						
	1	ATCC 17759†	ENV	Trinidad	Soil	1958
	1	LMG 14087	NON	UK	Wound	1988
	10	ATCC 25416^T^	ENV	USA	Onion	1948
	10	J1050	NON	USA	Human	Before 1983
	266	BC20	ENV	USA	Water	–
	266	AU6671	NON	U.S.	Wound	–
	365	HI-3602	ENV	USA	Soil	–
	365	C8509	CF	Canada	Sputum	1999
	365	AU3206	CF	USA	Sputum	–
*B.* multivorans						
	21	ATCC 17616†	ENV	USA	Soil	Before 1966
	21	AU0453	CF	USA	Sputum	–
	21	C9140	CF	Canada	Sputum	2000
	375	R-20526	ENV	Belgium	River water	2002
	375	IST455	CF	Portugal	Sputum	2000
*B.* cenocepacia IIIA						
	32	POPR8	ENV	Mexico	Radish	–
	32	5–457	CF	Czech Republic	Sputum	2002
	32	R-16597	CF	Belgium	Sputum	2001
	32	BCC1118	NON	UK	Wound	Before 1994
*B.* cenocepacia IIIB						
	37	BC-1	ENV	USA	Maize rhizosphere	–
	37	AU2362	CF	USA	Sputum	2000
	122	HI-2424	ENV	USA	Soil	–
	122	AU1054	CF	USA	CF	–
	122	CEP0497	NON	Canada	Leg ulcer	1995
*B.* stabilis						
	50	LMG 14294†	CF	Belgium	Sputum	1993
	50	R-16919	ENV	Belgium	Industrial	–
	50	LMG 14086†	ENVH	UK	Respirator	1970
	51	HI-2482	ENV	USA	Shampoo	–
	51	ATCC 35254	ENVH	USA	Medical solution	1980
	51	CEP0928	ENVH	USA	Albuterol solution	–
	51	LMG 14291	CF	Belgium	Sputum	1993
	51	LMG 7000	NON	Sweden	Blood	1983
*B.* vietnamiensis						
	61	J1702	ENVH	USA	Hospital equipment	–
	61	BCC0190	CF	USA	Sputum	–
	61	J1712	NON	USA	Wound	–
	61	J1738	NON	USA	Wound	–
	61	J1742	NON	USA	Wound	–
	67	R-20590	ENV	Belgium	River water	2002
	67	D0774	CF	Canada	Sputum	2003
B. ambifaria						
	81	MVP-C2-4	ENV	Italy	Maize rhizosphere	1996
	81	BCC0250†	CF	Australia	Sputum	1994
	77	AMMD^T^	ENV	USA	Soil	1985
	77	AU0212	CF	USA	Sputum	–

*MLST, multilocus sequence typing; Bcc, *B. cepacia* complex; ST, sequence type; ENV, isolated from the environment; NON, isolated from a non-cystic fibrosis (CF) patient; CF, isolated from a CF patient; IIIA or IIIB, isolates belonging to *B. cenocepacia recA* subgroup A or B, respectively; ENVH, isolated from a hospital environment. †, panel strain (*12*); suprascript T, type strain for species.

The resolution of strain identification offered by MLST is such that different isolates sharing the same ST (genotypically indistinguishable at all 7 loci) are defined as clones of the same strain (e.g., for a group of isolates within this collection, we have further validated this identity by cloning and sequencing up to 10 random fragments of DNA). The 15 STs identified from environmental and clinical sources belonged to 6 different Bcc species (Table): *B. cepacia* (4 STs), *B. multivorans* (2 STs), *B. cenocepacia* (3 STs), *B. stabilis* (2 STs), *B. vietnamiensis* (2 STs), and *B. ambifaria* (2 STs). Three *B. cenocepacia* STs also belonged to 2 different *recA* lineages defined within this species: IIIA (1 ST) and IIIB (2 STs).

## Conclusions

STs occurring in both clinical and environmental niches were found in 6 of the 9 formally described Bcc; the greatest degree of overlap occurred in *B. cepacia* and *B. stabilis* (Figure). The proportion of STs not shared between clinical and environmental isolates varied for each Bcc species we examined. This finding may reflect the few clinical or environmental isolates for that species or high genetic diversity; a larger sample size is needed to find identical matches. Species dominated by clinical STs (>83% of STs) were *B. multivorans, B. cenocepacia recA* lineage IIIA, and *B. dolosa.* Those species containing mainly environmental STs (>80%) were *B. ambifaria, B. anthina,* and *B. pyrrocinia* (Figure). Although this distribution agrees with findings of previous studies (*2*), it also reflects the distribution of isolates within the collections from which isolates were obtained.

**Figure:**
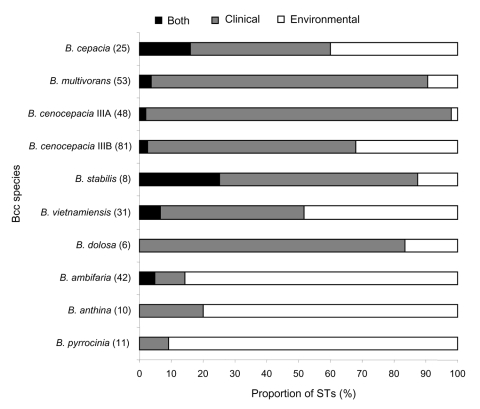
Proportion of sequence types (STs) within each *Burkholderia cepacia* complex (Bcc) species from clinical, environmental, or both sources. The bar chart shows the proportion of STs derived from the environment (white), clinical (gray), and both sources (black shading). The total number of STs examined for each *B. cepacia* species is in parentheses.

Several ST matches between clinical and environmental isolates were of particular interest. MLST ST-10 was shared by *B. cepacia* J1050, a human isolate cultured in the United States (Cleveland, Ohio) and the type strain for *B. cepacia* ATCC 25416, isolated from an onion. This evidence of clonality augments the clonal relationship reported earlier (*9*) between ATCC 25416 and a UK isolate from a CF patient. The *B. multivorans* IST455 isolated from a CF patient’s sputum in Portugal, as reported in a previous study (*13*) had the same sequence type (ST-375) as R-20526, which was isolated from the River Schelde in Belgium.

*B. cenocepacia recA* lineage IIIA isolates with ST-32 (Table) were from 4 independent sources: POPR8 (isolated from a radish in Mexico), BCC1118 (isolated from a UK non-CF patient infection), R-16597 (isolated from a CF patient in Belgium), and 5–457 (isolated from a CF patient in the Czech Republic). ST-32 appears to be a globally distributed, predominantly clinical strain (A. Baldwin, unpub. data). The *B. cenocepacia recA* lineage IIIB isolates identified as ST-122 (Table) include the PHDC strains, predominant in US CF patients (AU1054) and previously found in US soil (HI-2424) (*10*), and CEP0497, which was obtained from a leg wound in a non-CF patient in Canada. Together with a recent report of PHDC strains identified in Europe (*14*), the Canadian isolate in our study adds further weight to the identification of this ST as a globally distributed strain.

MLST analysis of *B. stabilis* corroborated the high degree of clonality observed by PFGE fingerprint analysis in the original description of this species (*15*). However, MLST was further able to distinguish 8 STs among the 26 isolates examined, which indicates that MLST may be a more effective method than PFGE for epidemiologic analysis of *B. stabilis*. This increased resolution adds to the observation that 2 *B. stabilis* STs are globally distributed and isolated from clinical samples and an array of different niches, including domestic products, medical solutions, industrial process contaminants, and hospital devices.

In summary, >20% of the clinical isolates we examined were indistinguishable by MLST from isolates from environmental sources. This finding suggests that conservation of intrinsic determinants necessary to thrive in environmental niches may confer an ability to colonize susceptible humans. Further work is urgently required to more extensively investigate the emergence of pathogenic members of the Bcc in the natural environment and the risk for infection this may represent.
